# Pupillary Measures of the Cognitive Effort in Auditory Novel Word Processing and Short-Term Retention

**DOI:** 10.3389/fpsyg.2018.02248

**Published:** 2018-11-27

**Authors:** Susana López-Ornat, Alexandra Karousou, Carlos Gallego, Leire Martín, Raquel Camero

**Affiliations:** ^1^Department of Psychology, Universidad Complutense de Madrid, Madrid, Spain; ^2^Department of Education Sciences in Early Childhood, Democritus University of Thrace, Komotini, Greece

**Keywords:** cognitive effort, task-evoked pupillary responses (TEPRs), auditory pseudo-words, novel word processing, phonological complexity, phonological short-term retention

## Abstract

The use of the task-evoked pupillary responses (TEPRs) methodology is emerging in the psycholinguistics literature, as a sensitive, reliable and dynamic psychophysiological measure of the cognitive effort produced by various aspects of language processing. This preliminary study aimed to assess the functionality and effectiveness of a TEPRs design for measuring the cognitive effort required for the processing and spontaneous (non-explicitly prompted) short-term retention of novel phonological forms presented auditorily. Twenty-four young adult participants (aged 19–28 years, *M* = 20.3, *SD* = 2.13) were auditorily presented with a series of pseudowords differing in their number of syllables and their syllabic complexity. Then, they were asked to produce a response to a delayed pseudoword–color matching task aimed to induce the short-term retention of the novel forms. Results on the size and timing of the TEPRs reveal a significant pupillary activation, starting immediately after the presentation of the auditory stimuli, peaking at 1080 ms and not subsiding significantly during the protracted retention period. Moreover, the differential complexity of the novel words phonology significantly affected pupillary activation. Overall, these preliminary results point to the effectiveness of pupillometry as a technique for capturing the cognitive effort entailed in the short-term maintenance of novel word forms in the phonological loop, a process deemed crucial in the everyday novel word learning process. Results are discussed in view of future research that could establish and extend their implications.

## Introduction

Task evoked pupillary responses (TEPRs) have been established as a sensitive and reliable psychophysiological measure of the cognitive effort entailed in a variety of tasks (Papesh and Goldinger, 2015). TEPRs provide rich information about the time-course of each processing event: they typically arise 200–400 ms after the onset of a triggering stimulus, peak within 1–2 s. and subside rapidly after termination of processing (Partala and Surakka, 2003). They are also sensitive to between-task, within-task and between-individual variations in cognitive effort (Beatty, 1982).

The use of the TEPRs is emerging in the psycholinguistics research (Schmidtke, 2017; Zekveld et al., 2018). The majority of relevant studies focus on the processing of *visually presented* linguistic stimuli (e.g., Cabestrero et al., 2009; Engelhardt et al., 2010; Papesh et al., 2012; Schmidtke, 2014; Guasch et al., 2016; Haro et al., 2016). The use of TEPRs in the study of *oral speech processing* is more recent. Relevant studies focus on speech listening effort in noisy contexts and degraded speech (e.g., Piquado et al., 2010; Koelewijn et al., 2012; Zekveld et al., 2013, 2014; Causse et al., 2016; McMahon et al., 2016; Wendt et al., 2017), on resolving ambiguous syntactic categories (Vogelzang et al., 2016), on semantic, syntactic, metric, or rhyme violations (Scheepers et al., 2012) or on the processing of difficult versus easy nouns (Chapman and Hallowell, 2015). To our knowledge, there is no TEPRs research on the cognitive effort required for the processing and short-term retention of auditory (pseudo)words.

This preliminary study aims to assess the effectiveness of a TEPRs design in measuring the cognitive effort required for the processing and spontaneous short-term retention of auditory novel words (pseudowords/PW). By that, we intend to capture the processing effort entailed in what is assumed to be a crucial initial step in the everyday word learning process (Baddeley et al., 1998). Our experimental design did not explicitly instruct participants to rehearse/repeat a PW. Instead, a delayed PW-color pseudotask made participants meet the need to maintain PW’s phonological representation for a time beyond the limits of short-term memory, inducing thus a more ‘ecologically valid’ rehearsal. This task closely resembles everyday novel word learning (matching a novel phonological representation with a meaning), with colors lacking a heavy semantic load which could potentially interfere.

Based on previous TEPRs findings, we hypothesized that a significant pupillary diameter (PD) increase should be produced after the PWs presentation, peak around 1–2 s., and be maintained during the entire retention period. Moreover, we hypothesized that, if PW rehearsal in the phonological loop is required for their protracted retention, then PWs of greater phonological complexity/difficulty would yield significantly larger TEPRs.

## Materials and Methods

This study was carried out in accordance with the ethical guidelines of the “Colegio Oficial de Psicólogos” (Spain) and approved by the Ethics Committee of the Universidad Complutense de Madrid. All participants gave their written informed consent in accordance with the Declaration of Helsinki.

### Participants

Twenty-four university students (18 female and 6 male) aged 19–28 years (*M* = 20.3, *SD* = 2.13) volunteered to participate in the study. Participants were right-handed, had normal vision and did not report any neurological, sensory, motor or behavioral problem/disability, nor a language impairment history.

### Stimuli

Six Spanish-sounding PWs -two disyllabic, two trisyllabic, two tetrasyllabic- were presented auditorily in a random order. Within each pair, one PW had a simple syllabic structure (no consonant clusters) and the other had a complex structure. PWs duration ranged 550–960 ms (Supplementary Material S1). The PWs were recorded by a female Spanish native speaker.

A set of 24 colors were selected to be non-prototypical tones, namely difficult to classify/name for Spanish speakers (Supplementary Material S2). Four colors were randomly assigned to each PW and combined in a slide (in vertical stripes). A stable luminance (100 ± 12 cd/m^2^) was ensured across the whole experiment.

### Apparatus and Arrangement

Participants were seated in a shielded Faraday cabin, on an ergonomic chair facing a table with a mouse placed within reach. PD of the participants’ right-eye was recorded by an ASL-Eye-Pupil-Tracker-504 Pan/Tilt Optics at 60 Hz (sampling period: 20 ms) positioned at a 70 cm distance. The PWs were reproduced by stereophonic speakers. A Casio XJ-A150V projected the visual stimuli on a Knox 200 Mercury screen (1.80 m × 1.65 m) located 2.30 m in front of the participants. Luminosity was constantly monitored using a Minolta CL-200 luxocolorimeter.

### Procedure

Participants received instructions concerning the task and the devices. They would hear six ‘weird’ fictitious words. Some seconds after the presentation of each word, a display of 4 colors would appear on the screen; their task would be to select –with a mouse-click - the color they considered best related to the word previously heard. It was emphasized there were no right or wrong answers. Calibration of the eye-tracker was performed according to the eye-tracker’s specifications.

The test phase started with summarizing the instructions on screen. Fifteen seconds later, a centered fixation point appeared to help participants focus attention and the PD started being recorded. The fixation point disappeared 500 ms later when a random PW was presented auditorily. The fixation point returned 1200 ms after the offset of the PW to help participants maintain attention during the remaining 2000 ms of the retention period, after which the color-slide appeared. Participants had to select one color with a mouse-click, after which (or automatically after 20 s) the procedure for the next PW presentation was initiated (see Supplementary Material S3).

### Data Selection and Definition

Pupillary diameter exit values in pixels were converted to millimeters according to the eye-tracker specifications. After a data cleaning/selection procedure, measures with anomalous pupillary size (<17 px/3.36 mm or > 40 px/7.92 mm) were eliminated. Overall invalid and missing values amounted to 4.05% across trials. After applying a linear interpolation procedure, a remaining 0.26% of missing points were excluded from analysis.

Data analyses were based on eleven sampling periods (Supplementary Material S3):

- [BL] Baseline: 400 ms preceding PW onset.
- [PW] PW auditory presentation (variable duration).
- [R1, R2, R3, R^+^1, R^+^2, R^+^2, R^+^4, R^+^5] Retention period devided in eight 400 ms periods.
- [C] 400 ms after color-slide onset (prior to color selection).

## Results

**FIGURE 1:**
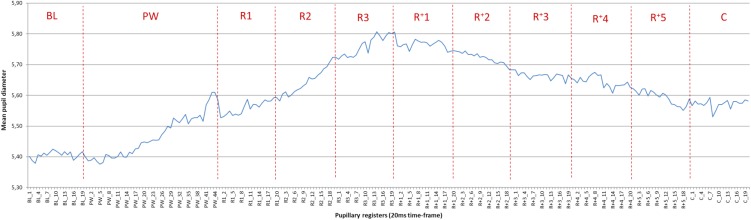
Mean pupil diameter across the task. Each point of the curve corresponds to the average value of all trials (six PWs) by the 24 participants (i.e., 144 observations). The curve at the time-points PW_1 to PW_28 reflects the average pupillary diameter (PD) for all six PWs, while after that point the values are based on the average PD of the remaining/longer words. For example, from PW_41 to PW_45 the scores correspond exclusively to the longest PW. Mean standard deviation across the task was 0.60 mm (min. = 0.45, max = 0.73).

Figure 1 represents the mean PD values per 20 ms. PD increases progressively from BL (5.41 mm) to peak during the last half of the *R3*-period. Then the activation progressively decreases, although during the analyzed period (*before* selecting a color) it does not return to *BL* value.

*Mean Peak Diameter* (5.81 mm) is produced at 1060–1080 ms after PW offset (*Peak Latency*). This value represents an increment of 0.40 mm with respect to *BL* (5.41 mm), which corresponds to a *Mean Peak Dilation* of 7.39%.

To explore significant differences between the BL and mean PDs, mean values have been calculated on a sampling period basis (see, Table 1).

**Table 1:** Mean pupil diameter values (mm), Peak Diameter (mm), Peak Latency (ms), and Peak Dilation (% of increment with respect to the mean BL) per sampling period.

Sampling period	Mean pupil diameter (SD)	Peak Diameter (SD)	#Measure	Peak Latency (ms)	Peak Dilation (%)
BL	5.41 (0.60)	5.43 (0.62)	BL_9		
PW	5.47 (0.60)	5.61 (0.59)	PW_43		
R1	5.56 (0.61)	5.59 (0.63)	R1_19	360	2.95
R2	5.65 (0.63)	5.72 (0.63)	R2_19	760	5.73
R3	5.76 (0.61)	5.81 (0.61)	R3_14	1080	7.39
R^+^1	5.76 (0.66)	5.78 (0.68)	R^+^1_7	1340	6.83
R^+^2	5.72 (0.66)	5.74 (0.66)	R^+^2_1	1600	6.10
R^+^3	5.66 (0.65)	5.69 (0.65)	R^+^3_1	2000	5.18
R^+^4	5.64 (0.65)	5.65 (0.65)	R^+^4_1	2400	4.44
R^+^5	5.59 (0.66)	5,62 (0.66)	R^+^5_1	2800	3.88
C	5.56 (0.68)	5.59 (0.67)	C_7	2920	3.33


The mean PD starts to increase immediately after the presentation of the PWs. The maximum mean PD is reached during the *R3-*period and is maintained for the *R^+^1-*period. However, the Peak Diameter value (the highest value recorded within each period) is higher for the *R3*-period, as at 1080 ms after the offset of the PWs the pupil reaches its highest diameter (5.81 mm). From the *R^+^2*-period on, PD gradually decreases, and during the *C-*period (400 ms with color-screen) the pupil remains 3.33% larger than *BL*.

Paired-samples *t*-tests (Bonferroni-corrected significance threshold *p* < 0.0011) revealed significant differences between the mean PD and *BL* in all periods (*p* = 0.000) with the exception of the *PW*-period (*p* = 0.046) and *C*-period (*p* = 0.003) Pupillary activation is, thus, already significant in the R1-period (immediately after the PWs offset) and maintained during the entire retention period.

The mean PD of the *R^+^1*-period is significantly larger than in all other periods (*p* < 0.001), with the exception of the preceding *R3* (*p* = 0.992) and the subsequent *R^+^2* (*p* = 0.236). Thus, a group of maximum activation, formed by the periods *R3*, *R^+^1* and *R^+^2*, can be deduced. It is reached gradually and, later on, is gradually reduced.

In order to address the possibility of PD not returning to *BL*-value after the *C*-period, paired-samples *t*-tests (Bonferroni-corrected significance threshold *p* < 0.003), comparing the mean *BL* of each trial with the mean *BL* of the immediately next trial, revealed a non-significant decrease in the BL values (*p* > 0.083 in all cases; Mean BL per trial: BL1 = 5.54 mm, BL2 = 5.53 mm, BL3 = 5.43 mm, BL4 = 5.37 mm, BL5 = 5.34 mm, BL6 = 5.26 mm) suggesting that return to BL is reached at some point after the completion of the task (color selection).

Finally, assessing possible modulation of pupilary activation by the phonological complexity of the PWs, repeated-measures ANOVAs revealed a significant effect of “*syllabic complexity”* [*F*(1,179) = 1253.32, MSE = 2.32, *p* < 0.001, 1-β = 1, $\eta_{p}^{2}$ = 0.87] with complex PWs producing larger PDs and with a very large effect size, and “*number of syllables”* [*F*(2,178) = 7.18, MSE = 0.033, *p* < 0.001, 1-β = 0.93, $\eta_{p}^{2}$ = 0.16] with tetrasyllabic PWs producing larger PDs, and with a medium effect size (Supplementary Materials S4, S5).

## Discussion

Results support our initial hypotheses: a cognitive effort [quantified by significant PD increases compared to *BL*] was evident from the PWs offset, peaked 1080 ms later and slowly decreased thereafter. Unlike previous results on oral language processing -without a delayed task (e.g., Mathôt et al., 2017), PD did not subside rapidly after peak; it remained significantly higher than *BL* during the entire retention period (3200 ms). Then, during the 400 ms after the color-screen presentation, the earliest difference of mean PD to *BL* was recorded, even though PD still remained 3.33% larger than *BL*.

Due to technical constraints (time-locked measurements only), we haven’t been able to analyze the TEPRs produced at and immediately after the completion of the mental task (color-selection) as its timing varied among participants/trials. Nonetheless, comparisons between each trial’s *BL* and the *BL* of the subsequent trial yielded a decreasing pattern providing additional evidence of the pupil’s return to *BL* after termination of processing (Beatty and Lucero-Wagoner, 2000; Partala and Surakka, 2003).

Moreover, results revealed a significant effect of the factors ‘syllabic complexity’ and ‘number of syllables’ on the TEPRs, with more complex and long pseudowords yielding greater pupillary activation.

We assume these results successfully captured the protracted effort produced presumably to retain through subvocal rehearsal the PW-forms for a period beyond the 1–2 s. time-limit of the phonological store (Baddeley, 2012), in order to use them in the delayed PW-color matching task, i.e: a word-learning relevant task.

## Limitations and Future Directions

In this preliminary study we identified some limitations which need to be addressed in view of subsequent studies. An improvement of the design is needed so that direct evidence of the PD return to *BL* after the termination of processing can be captured. An event-locked measurement of PD immediately after completion of the task should be introduced.

Moreover, the PD amount that is attributed to the retention effort from that caused by the phonological processing of the stimuli could be disentangled. A new design could add a control condition where participants would just be auditorily presented with the same PWs without being induced to retain them thereafter. Alternatively, a control condition where participants would be explicitly prompted to repeat/rehearse PWs after the same amount of time would further strengthen this research line.

Finally, a full-scale study is planned to include more trials per condition (presenting more PWs per number of syllables/syllabic complexity). This will permit more in-depth analyses on the effect of the PWs phonological complexity on the effort required for their retention.

## Conclusion

Overall, these results point to the effectiveness of pupillometry in capturing the cognitive effort entailed in the processing and short-term retention of auditorily presented novel words. This first attempt successfully captured TEPR indices of a sustained effort which, additionally, appeared to be modulated by the complexity of the phonological forms. This protracted effort can be attributed to subvocal rehearsal processes that are deemed necessary for the maintenance of phonological information beyond the 1–2 s time-limit of the phonological store (Baddeley, 2012). Various studies already attributed a very important role to these processes for word learning (e.g., Baddeley, 2003; Gathercole, 2006). But, to our knowledge, this is the first time the cognitive effort involved has been shown to be psychophysiologically measurable with TEPRs. We believe this study constitutes a first step into a novel line of TEPRs research aimed at establishing interesting relationships between the magnitude/nature of the mental effort allocated by each individual and his/her ability for novel word learning.

## Author Contributions

AK and SL-O conceived and designed the study. SL-O and LM prepared the experimental materials and set-up. RC, LM, and SL-O collected the data. RC and LM prepared the database for analysis and formatted the reference list. CG analyzed the data. CG, AK, and SL-O interpreted the results and drafted the manuscript. SL-O coordinated the project. All authors approved the version to be published.

## Conflict of Interest Statement

The authors declare that the research was conducted in the absence of any commercial or financial relationships that could be construed as a potential conflict of interest.
