# Supplementary figures and images for: Pupillary Measures of the Cognitive Effort in Auditory Novel Word Processing and Short-Term Retention
Source: Front Psychol. 2018 Nov 27;9:2248. doi: 10.3389/fpsyg.2018.02248 (PMC6278650; doi:10.3389/fpsyg.2018.02248)

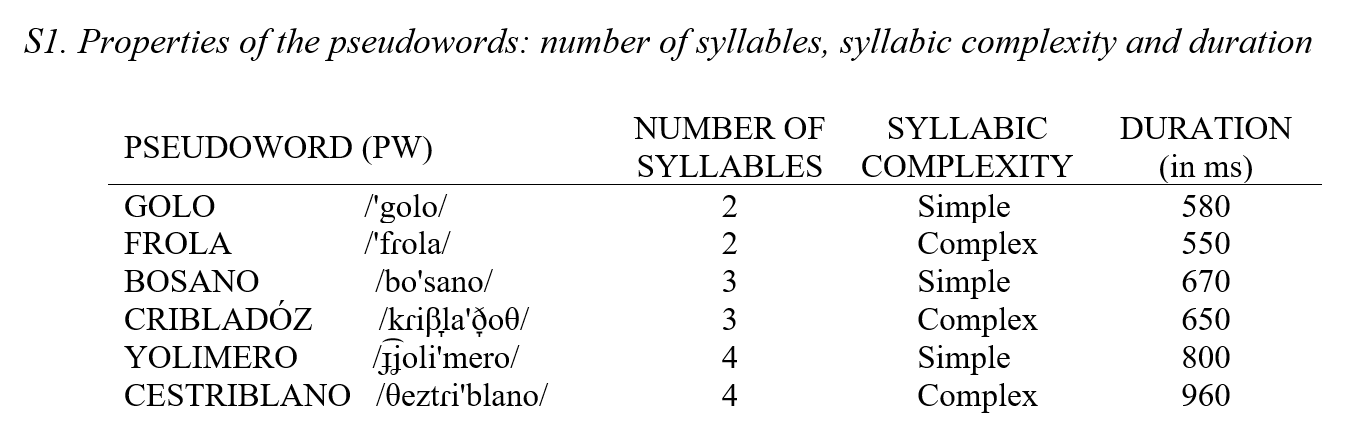

Supplement: Supplementary file 1 [file Image_1.png]

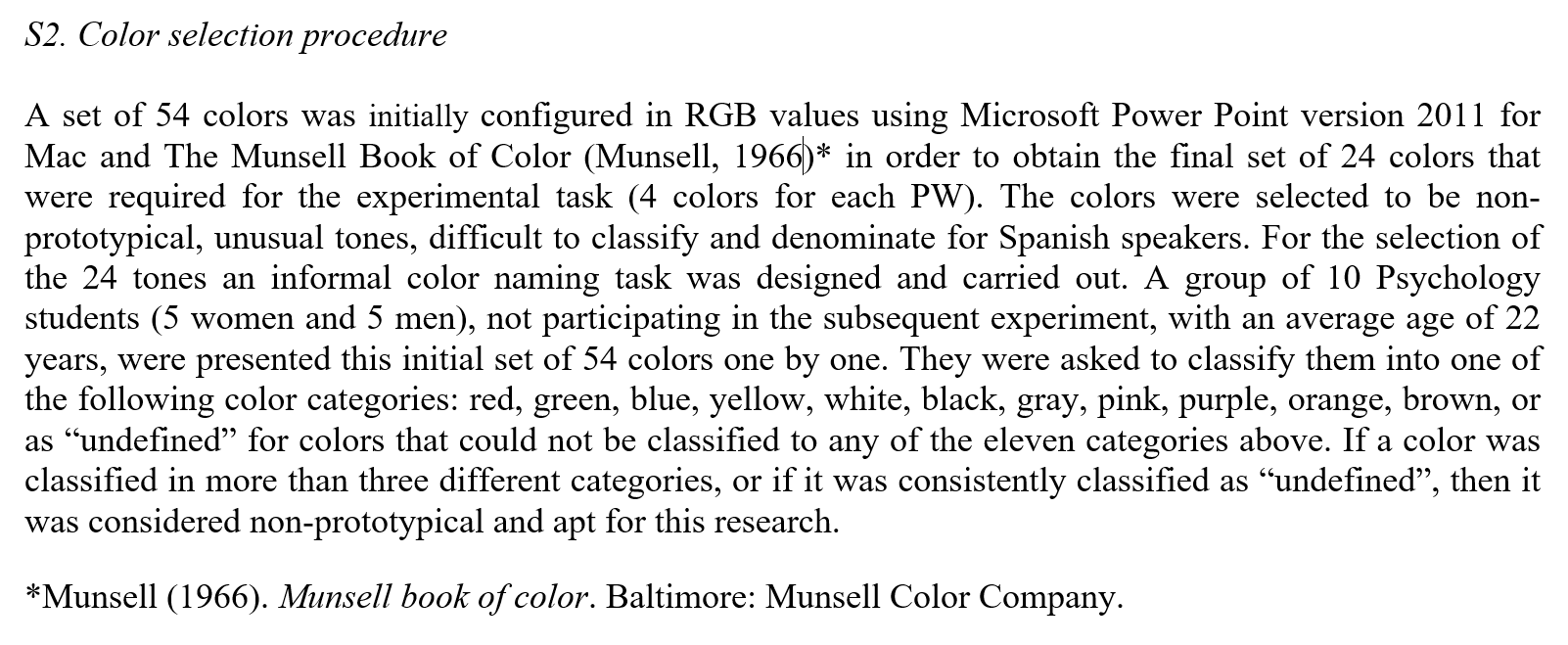

Supplement: Supplementary file 2 [file Image_2.png]

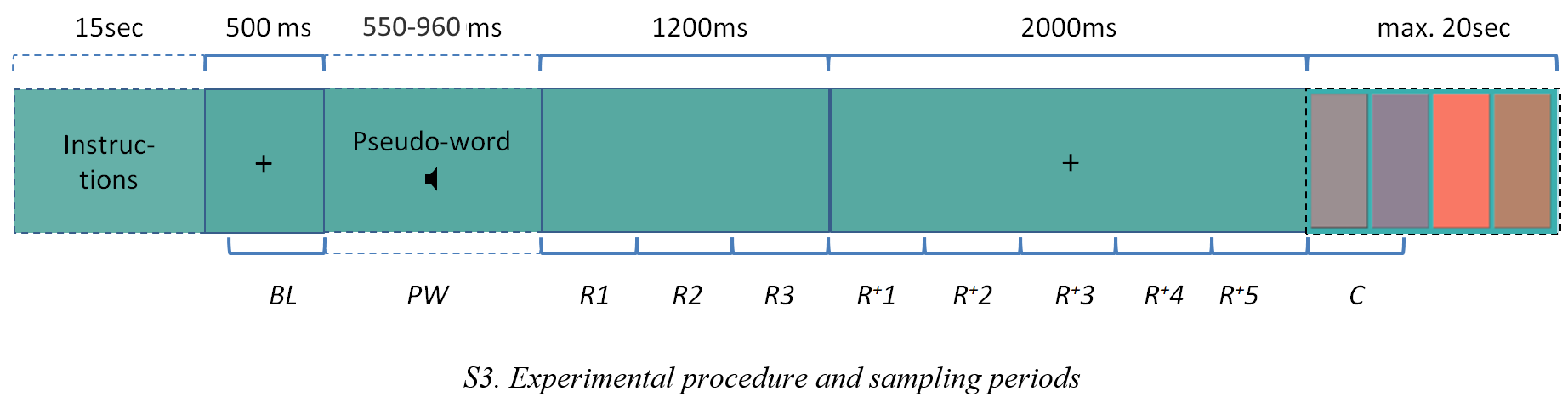

Supplement: Supplementary file 3 [file Image_3.png]

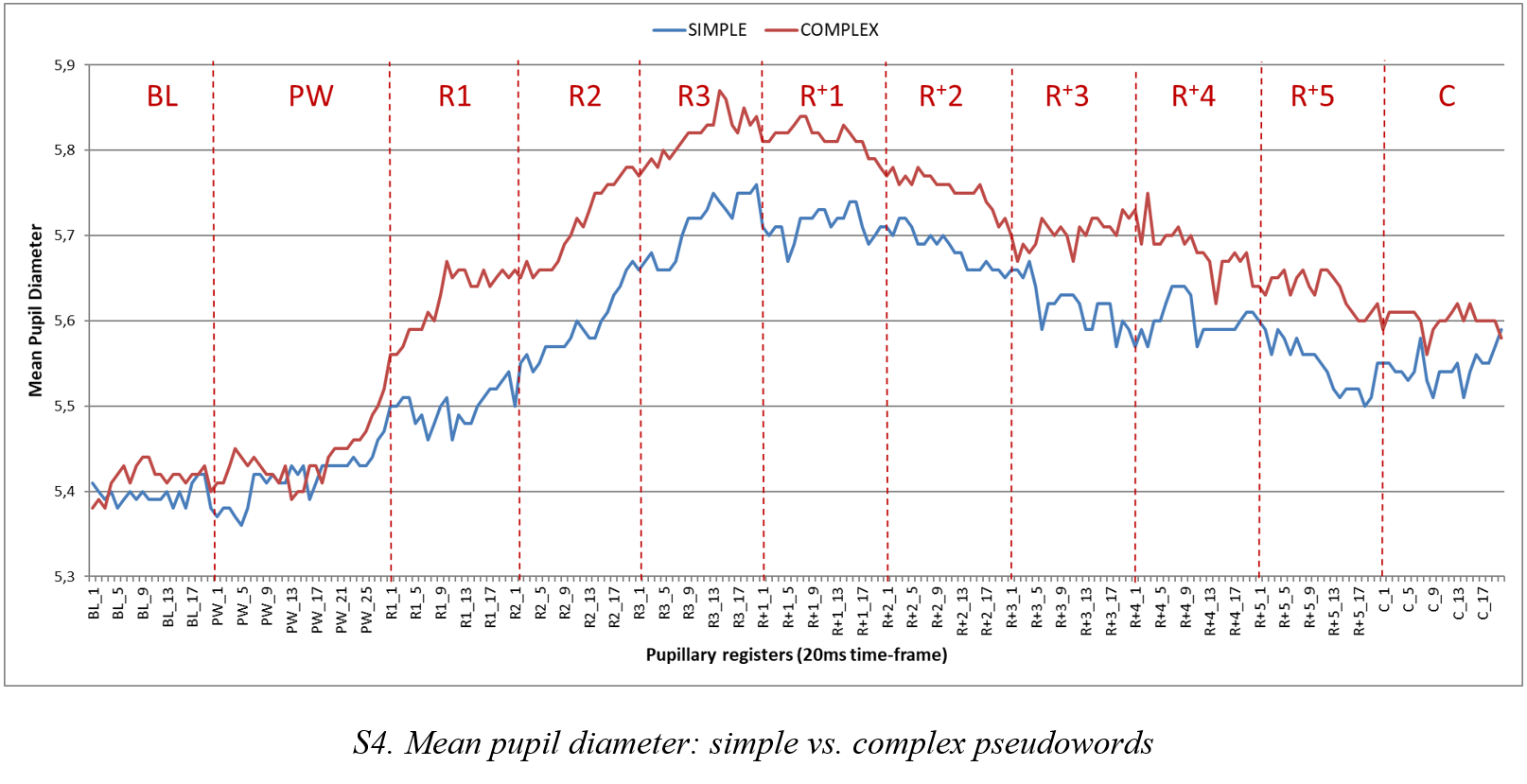

Supplement: Supplementary file 4 [file Image_4.png]

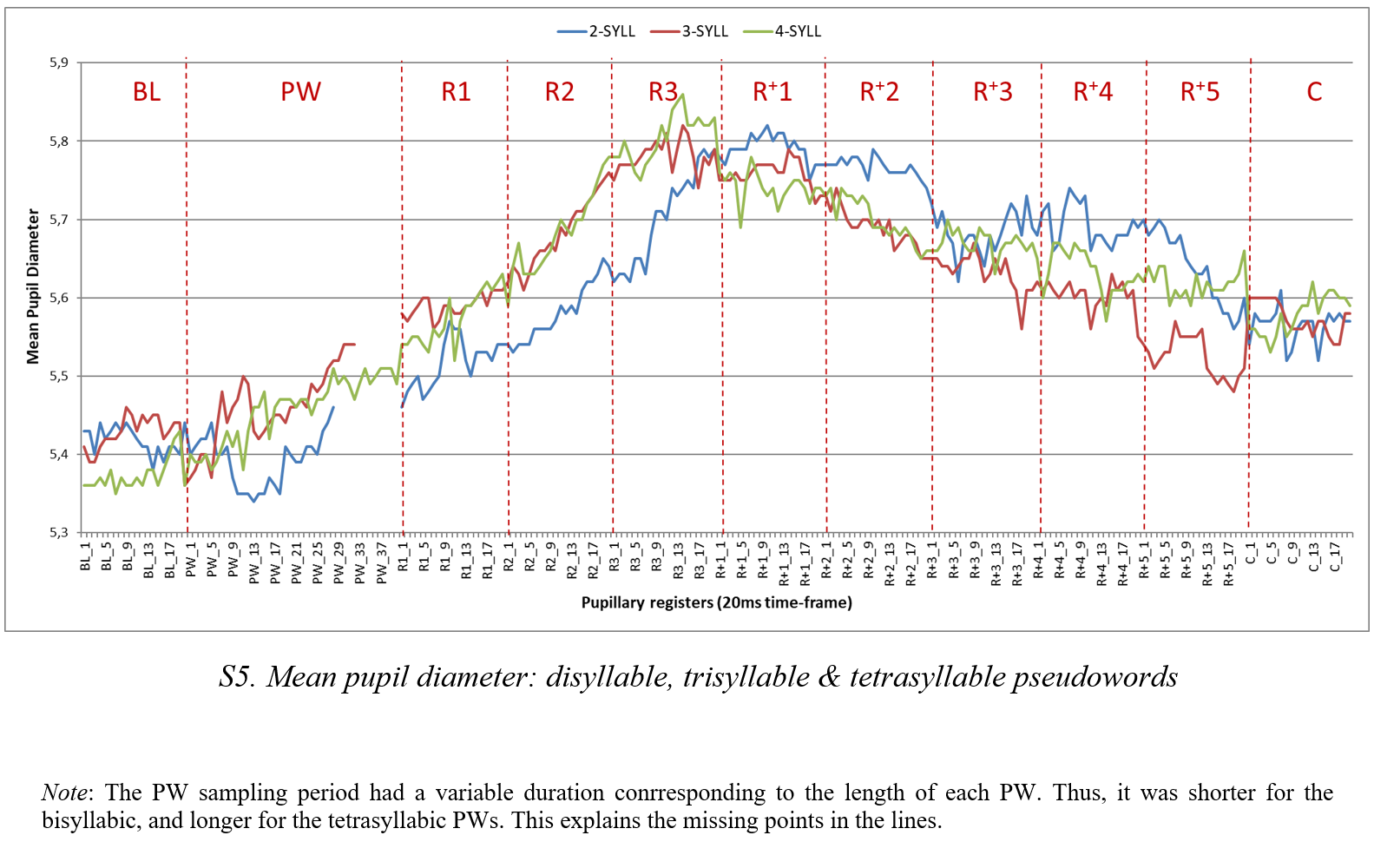

Supplement: Supplementary file 5 [file Image_5.png]
